# Graphene growth from reduced graphene oxide by chemical vapour deposition: seeded growth accompanied by restoration

**DOI:** 10.1038/srep22653

**Published:** 2016-03-10

**Authors:** Sung-Jin Chang, Moon Seop Hyun, Sung Myung, Min-A Kang, Jung Ho Yoo, Kyoung G. Lee, Bong Gill Choi, Youngji Cho, Gaehang Lee, Tae Jung Park

**Affiliations:** 1 Department of Chemistry, Chung-Ang University, 84 Heukseok-ro, Dongjak-gu, Seoul 06974, Republic of Korea; 2 Measurement & Analysis Team, National Nanofab Center, 291 Daehak-ro, Yuseong-gu, Daejeon 305-701, Republic of Korea; 3 Thin Film Materials Research Center, Korea Research Institute of Chemical Technology, 141 Gajeong-ro, Yuseong-gu, Daejeon 305-600, Republic of Korea; 4 Department of Nano Bio Research, National Nanofab Center, 291 Daehak-ro, Yuseong-gu, Daejeon 305-701, Republic of Korea; 5 Department of Chemical Engineering, Kangwon National University, 346 Joongang-ro, Samcheok 245-711, Republic of Korea; 6 Department of Applied Science, Korea Maritime and Ocean University, Busan 606-791, Republic of Korea; 7 Korea Basic Science Institute, 169-148 Gwahang-ro, Yuseong-gu, Daejeon 305-806, Republic of Korea

## Abstract

Understanding the underlying mechanisms involved in graphene growth via chemical vapour deposition (CVD) is critical for precise control of the characteristics of graphene. Despite much effort, the actual processes behind graphene synthesis still remain to be elucidated in a large number of aspects. Herein, we report the evolution of graphene properties during in-plane growth of graphene from reduced graphene oxide (RGO) on copper (Cu) via methane CVD. While graphene is laterally grown from RGO flakes on Cu foils up to a few hundred nanometres during CVD process, it shows appreciable improvement in structural quality. The monotonous enhancement of the structural quality of the graphene with increasing length of the graphene growth from RGO suggests that seeded CVD growth of graphene from RGO on Cu surface is accompanied by the restoration of graphitic structure. The finding provides insight into graphene growth and defect reconstruction useful for the production of tailored carbon nanostructures with required properties.

The growth of graphene on copper (Cu) substrates via chemical vapour deposition (CVD)^1^,^2^ has been extensively exploited for the purpose of achieving large-area, high-quality single crystals, which are highly desirable for the practical use of graphene in industrial applications^3^,^4^,^5^,^6^. Together with their technological appeal, such systems also serve as a unique platform for broadening our fundamental understanding of a new and intriguing class of growth phenomena. In particular, the overall properties of CVD-grown graphene films are sensitively dependent on diverse parameters^7^,^8^,^9^,^10^,^11^,^12^ including purity of copper, types of carbon precursors, temperature, and vapour pressure. However, the wide variation in properties of CVD-grown graphene films under similar growth conditions suggests that fine-tuning of the growth parameters is still required. Thus, the actual processes and the underlying mechanisms involved in graphene growth^7^,^8^,^9^,^10^,^11^,^12^,^13^,^14^,^15^ are vital to understand for achieving precise control of the graphene growth.

CVD growth of graphene on Cu is a surface-mediated process^14^. During the CVD process, nucleation of graphene critical nuclei occurs spontaneously and randomly on the Cu surface, and then monolayer graphene is subsequently synthesized from the edge of the graphene nuclei^13^,^14^,^15^,^16^. Recently, monolayer graphene has been also grown from seeds intentionally patterned or prepared on Cu prior to the CVD process^16^,^17^,^18^,^19^, instead of from graphene seeds spontaneously and randomly nucleated on Cu during the CVD process. Specifically, CVD-grown graphene monolayer or multilayer grains^17^,^18^ and mechanically exfoliated graphene or graphite flakes^17^,^18^ have been utilized as seeds for obtaining high-quality monolayer graphene. In addition, poly(methyl methacrylate) (PMMA) dots^19^ and chemically derived graphene oxide (GO) flakes^20^ have been also used for seeded CVD growth of high-quality monolayer graphene. However, complete restoration of graphitic structure in chemically derived GO by a reduction process remains a considerable challenge^21^. In practice, chemically derived GO or even its reduced form exhibits highly defective graphene structures^22^,^23^ compared with CVD-grown or mechanically exfoliated graphene and PMMA at high temperature^24^. Additionally, reduced graphene oxide (RGO) flakes on silicon dioxide (SiO_2_) surfaces serve as templates for the new growth of defective graphene during ethanol CVD^25^. Accordingly, a detailed understanding of the growth of high-quality graphene from RGO flakes on Cu during the CVD process remains to be elucidated.

Here we report the variation of graphene properties during lateral growth of graphene from RGO flakes on polycrystalline Cu foils by methane CVD. A combined microscopic and spectroscopic study correlated the growth length of CVD-grown graphene from RGO, reflecting the stages of in-plane graphene growth, with the corresponded structural quality of the graphene. The correlation demonstrated that graphene exhibited substantial enhancement in structural quality while it was laterally grown from RGO flakes on Cu surfaces up to a few hundred nanometres by the CVD process. The monotonous improvement of the structural quality of the graphene with increasing extended length of the graphene grown from RGO suggested that seeded CVD growth of graphene from RGO as low-quality seeds on Cu substrates was accompanied by the restoration of graphitic structure.

## Results

### Seeded CVD growth of graphene from RGO on Cu

**Figure 1:**
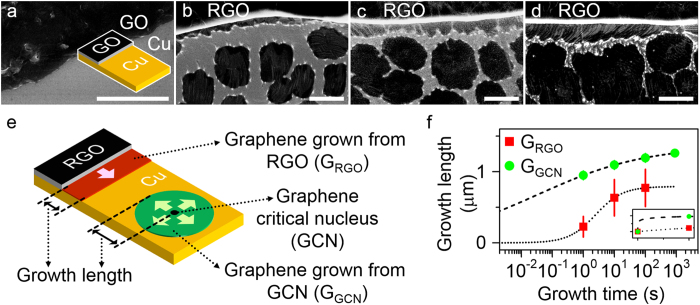
CVD growth of graphene on Cu with RGO. (**a**) SEM image of a region near GO edges on Cu before CVD. (**b–d**) SEM images of regions near RGO edges on Cu after CVD for (**b**) 1 s, (**c**) 10 s and (**d**) 100 s, showing the evolution of seeded CVD growth of graphene with time. The scale bars in (**a–d**) are 2 μm. (**e**) Schematic illustration of seeded CVD growth of graphene areas simultaneously grown from two types of seeds (RGO and GCN) on Cu. RGO was intentionally prepared on the Cu before seeded CVD growth, whereas GCN was spontaneously nucleated on the same Cu during CVD process before the onset of seeded CVD growth of graphene. (**f**) Evolution of the average growth length with time.

Initially, CVD growth of graphene was investigated on the Cu substrate seeded with GO flakes to confirm and characterize seeded CVD growth of graphene from RGO on Cu. To this end, graphene samples synthesized on Cu foils with GO flakes by CVD for several growth times (see Methods) were directly measured using a scanning electron microscope (SEM). The GO flakes, instead of RGO flakes, were prepared on Cu foils before CVD because they were naturally reduced (Supplementary Fig. S1) upon heating to achieve the CVD growth temperature^20^. SEM images (Fig. 1a–d) presented a region near the edge of GO flakes on Cu before CVD and after CVD for 1, 10 and 100 s, respectively. Prior to the beginning of the CVD process, no feature distinct from GO flakes on the Cu was observed at the edge of the GO flakes (Fig. 1a). After CVD growth for 1 s, however, a ribbon-like graphene confirmed by Raman spectroscopy (Supplementary Fig. S2) newly appeared along edges of RGO flakes on the Cu substrate (Fig. 1b). In addition, the growth front of the ribbon-like graphene moved in a direction away from RGO edges as CVD growth time increased (Fig. 1c,d). These results suggested the seeded CVD growth of graphene from RGO on the Cu, indicating that the RGO played a role as seeds exhibiting low-quality graphene structures. Under our CVD conditions, graphene islands were also observed near the ribbon-like graphene and their size also increased with the increase of CVD growth time (Fig. 1b–d). Unlike the ribbon-like graphene, graphene grains similar in size and shape to the graphene islands in Fig. 1b–d were also observed on Cu foils not seeded with RGO flakes after CVD under the same condition (Supplementary Fig. S3). These observations implied that the graphene critical nuclei (GCN) were spontaneously nucleated on Cu surfaces and then graphene islands were subsequently grown from such GCN as seeds exhibiting high-quality graphene structures during the CVD process^13^,^16^,^26^,^27^. For clarity, the graphene specimens grown from RGO and GCN on the Cu by CVD are referred to as G_RGO_ (Fig. 1e, red area) and G_GCN_ (Fig. 1e, green area), respectively.

Graphene edge structures can govern the kinetics of graphene growth^28^. However, effects of the structural quality of seeds on the kinetic behaviour of the CVD growth from graphene seeds are nearly unclear. In order to understand such effects, the size evolution of G_RGO_ and G_GCN_ on the same Cu surface with the increase of CVD growth time was examined and compared. The G_RGO_ growth length was defined as the distance between the G_RGO_ growth front and the corresponding RGO edge (Fig. 1e), and its values were directly measured from SEM images (Supplementary Fig. S4). Unlike G_RGO_, the growth length of G_GCN_ was difficult to measure directly from SEM images because of frequent coalescence between individual G_GCN_ islands after CVD for growth times longer than 10 s (Fig. 1d). Hence, the growth length of G_GCN_ was theoretically estimated using the experimentally-measured G_GCN_ coverage on the Cu foil with the corresponding GCN density based upon a simple model (Supplementary Fig. S6). In the model, individual G_GCN_ islands were approximated as identical circles for calculation of average growth length. The average growth length of G_RGO_ (Fig. 1f, red square) and G_GCN_ (Fig. 1f, green circle) was shown as a function of growth time together with a best-fit curve to the experimental data of G_RGO_ (Fig. 1f, dotted line, Supplementary Fig. S5) and G_GCN_ (Fig. 1f, dashed line, Supplementary Fig. S6c). The average growth length of G_RGO_ after CVD for 1 s was nearly 4 times shorter than that of G_GCN_ near G_RGO_, indicating that G_RGO_ was slowly formed during the early stages of growth compared with G_GCN_ (Fig. 1f, inset). Specifically, G_RGO_ and G_GCN_ took approximately 4 and 0.004 s, respectively, until the growth length reached nearly 500 nm in the early stages of growth (Fig. 1f, dotted and dashed lines). In addition, the area of G_RGO_ was also smaller than that of G_GCN_ as the nearest neighbour (Supplementary Fig. S7), implying that the seeded CVD growth of G_RGO_ on Cu surfaces was not preferred compared with that of G_GCN_ on the same Cu surface in the initial stages. According to previous studies^16^,^17^,^18^,^19^, however, seeded CVD growth of graphene from high-quality graphene seeds intentionally placed on Cu foils before CVD process is preferred compared with that of graphene from spontaneously nucleated GCN on the same Cu foil. Thus, we suggest that the low-quality graphene structures of RGO flakes resulted in the slow seeded CVD growth from the RGO flakes on the Cu as compared with the graphene CVD growth from GCN as seed crystals on the same Cu in the initial stages.

### Structural quality of graphene grown from RGO

**Figure 2:**
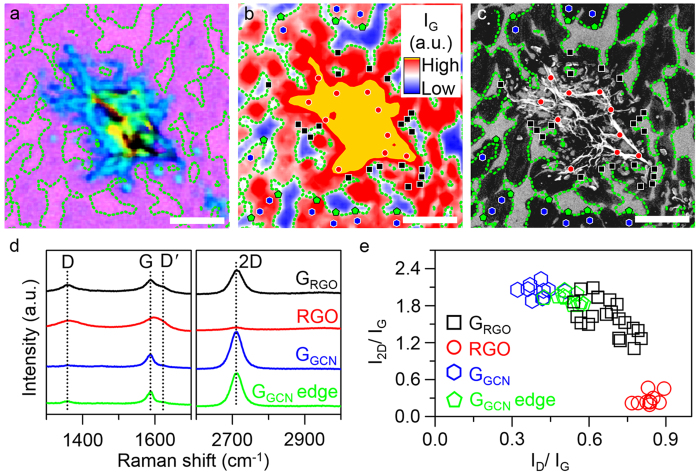
Structural characteristics of graphene formed during the initial stages of CVD growth on the Cu with RGO. (**a**) Optical microscope image, (**b**) Raman map of the G peak intensity and (**c**) SEM image of the same area of graphene grown on the Cu with RGO after CVD for 5 s and then transferred onto SiO_2_/Si. The perimeter of CVD-grown graphene on SiO_2_/Si was indicated by green-dotted line in (**a–c**). The scale bars in (**a–c**) are 3 μm. (**d**) Representative Raman spectra of G_RGO_, RGO, G_GCN_ and G_GCN_ edges in (**a–c**). (**e**) Correlation between I_D_/I_G_ and I_2D_/I_G_. The values of I_D_/I_G_ and I_2D_/I_G_ in (**e**) were calculated from Raman spectra measured at positions indicated by open black square (G_RGO_), open red circle (RGO), open blue hexagon (G_GCN_) and open green pentagon (G_GCN_ edges) in (**b,c**).

Graphene edge structures can govern the structural properties of subsequently grown graphene from the graphene edge structures^29^,^30^,^31^, implying that the low quality of RGO flakes may affect the structural quality of G_RGO_. Raman spectroscopy is suited to obtaining information concerning the number of graphene layers^32^ and identifying the presence of defects^33^,^34^,^35^,^36^. In order to understand the structural quality of G_RGO_ exhibiting slow kinetic behaviour in the early stages of growth, Raman spectroscopy was performed on graphene samples grown on the Cu with GO flakes by CVD for 5 s and then transferred onto a SiO_2_ layer on the silicon (Si) substrate (SiO_2_/Si, Fig. 2a). Especially, Raman map of the G peak intensity (I_G_, Fig. 2b) and SEM image (Fig. 2c) over the same area of the graphene sample were used for a precise spatially-resolved Raman spectroscopy of the graphene sample. As shown in Fig. 2b,c, Raman spectra of G_RGO_ (black square), RGO (red circle), G_GCN_ (blue hexagon) and G_GCN_ edges (green pentagon) were measured at several positions on the sample. Thick RGO (bluish, greenish and yellowish areas) and thin CVD-grown graphene (purplish area) on the SiO_2_/Si (pink area) were clearly discernible in an optical microscope image^37^,^38^,^39^ (Fig. 2a). For clarity, the boundary between the thin CVD-grown graphene and the bare SiO_2_/Si substrate was indicated by a green-dotted line in Fig. 2a–c. The D, G and 2D peaks were prominent in a representative Raman spectrum of G_RGO_ (Fig. 2d, black trace). Moreover, the D’ peak was also discernible as a weak shoulder peak of the G peak. Compared with G_RGO_, RGO (Fig. 2d, red trace) and G_GCN_ (Fig. 2d, blue trace), including G_GCN_ edges (Fig. 2d, green trace), exhibited extremely weak intensities of the 2D and D peaks, respectively, in their representative Raman spectra. In particular, the intensity ratio of the D peak to the G peak (I_D_/I_G_) of G_RGO_ varied widely from 0.55 to 0.80 (Fig. 2e, open black square) as compared with those of RGO (0.76–0.89, Fig. 2e, open red circle), G_GCN_ (0.32–0.50, Fig. 2e. open blue hexagon) and G_GCN_ edges (0.42–0.57, open green pentagon). The I_D_/I_G_ values of G_RGO_ were mostly larger than those of G_GCN_ and G_GCN_ edges, whereas these values were substantially smaller than those of RGO, indicating that the structural quality of G_RGO_ was lower than that of G_GCN_ and G_GCN_ edges, but it was higher than that of RGO. Similar to the distribution of the I_D_/I_G_ values, the intensity ratio of the 2D peak to the G peak (I_2D_/I_G_) of G_RGO_ also scattered over relatively wide ranges compared with those of RGO, G_GCN_ and G_GCN_ edges (Fig. 2e). The I_2D_/I_G_ values of G_RGO_ were substantially smaller than those of G_GCN_ and G_GCN_ edges, whereas these values were appreciably larger than those of the RGO flakes. Moreover, I_2D_/I_G_ of G_RGO_ tended to decrease with increasing I_D_/I_G_ of G_RGO_ (Fig. 2e, open black square). The average I_2D_/I_G_ values of G_GCN_ and G_GCN_ edges were 2.03 ± 0.10 (Fig. 2e, open blue hexagon) and 1.91 ± 0.08 (Fig. 2e, open green pentagon), respectively, indicating that G_GCN_ was a monolayer of graphene^40^. In addition, the colour of G_RGO_ in the optical microscope image^37^,^38^,^39^ (Fig. 2a) and the contrasts of G_RGO_ in the SEM image^41^ (Fig. 2c) were not distinguishable from those of G_GCN._ Thus, we suggested that the lower structural quality of G_RGO_ formed during the early stages of seeded CVD growth resulted in the average I_2D_/I_G_ values of G_RGO_ (1.62 ± 0.28) substantially smaller those of high-quality graphene monolayer^40^. However, the origin of the tendency in correlation between I_D_/I_G_ and I_2D_/I_G_ of G_RGO_ has yet to be specified.

### Evolution of the structural quality of graphene grown from RGO

**Figure 3:**
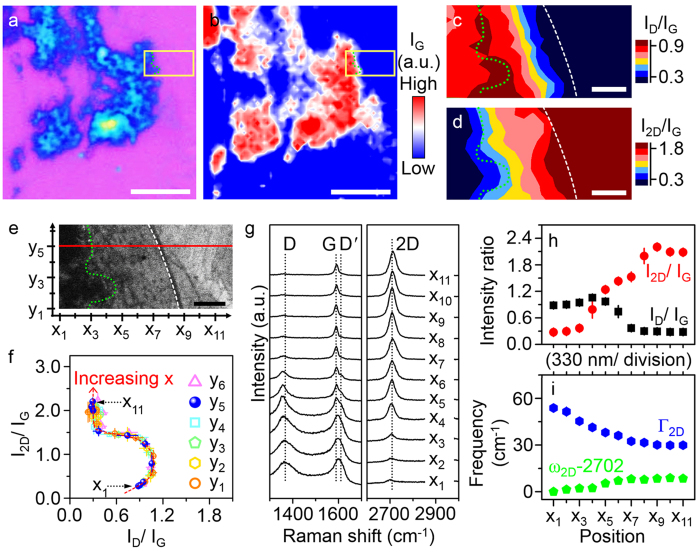
Evolution of structural characteristics of graphene formed during the later stages of CVD growth on the Cu with RGO. (**a**) Optical microscope image and (**b**) Raman map of I_G_ of the same area of graphene grown on the Cu with RGO after CVD for 900 s and then transferred onto the SiO_2_/Si. Optical characterization of the graphene sample on a large scale demonstrated that CVD-grown graphene completely covered the areas between RGO flakes, suggesting that G_RGO_ and G_GCN_ coalesced into a single large-area graphene film (Supplementary Fig. S8). The scale bars in (**a,b**) are 5 μm. (**c**) Raman map of I_D_/I_G_, (**d**) Raman map of I_2D_/I_G_ and (**e**) SEM image of the same area indicated by yellow box in (**a,b**). The perimeter of thick RGO and the line feature in (**e**) were indicated by green-dotted line and white-dashed line, respectively, in (**c–e**). The scale bars in (**c–e**) are 660 nm. (**f**) Correlation between I_D_/I_G_ and I_2D_/I_G_, showing that I_D_/I_G_ varied non-monotonously with the position movement from thick RGO (the left end in (**c–e**)) to high-quality monolayer graphene (the right end in (**c–e**)), whereas I_2D_/I_G_ increased monotonously. The data in (**c,d**) were used as the values of I_D_/I_G_ and I_2D_/I_G_ in (**f**). (**g**) Raman spectra as a function of position. The Raman spectra in (**a**) were obtained along the red-solid line in (**e**). (**h**) I_D_/I_G_ (black sqaure) and I_2D_/I_G_ (red circle) and (**i**) ω_2D_ (green pentagon) and Γ_2D_ (blue hexagon) as a function of position. The values of the Raman characteristic parametres including I_D_/I_G_, I_2D_/I_G_, ω_2D_ and Γ_2D_ in (**b,c**) were calculated from the Raman spectra in (**a**).

Although the low structural quality of the initially-formed G_RGO_ during seeded CVD growth has been specified, little is known about the evolution of the structural quality of graphene subsequently grown from the initially-formed G_RGO_ during the later stages of CVD growth. To understand the evolution of the structural quality of G_RGO_ with the increase of growth time, we measured Raman spectra on graphene samples in a direction away from the RGO flakes because the growth front of G_RGO_ moved with time in such a direction during the CVD process, as already demonstrated in Fig. 1. To obtain the maximum growth length of G_RGO_, the graphene samples were grown on the Cu with GO flakes by CVD for 900 s. Large-scale characterization^42^,^43^,^44^,^45^ of the graphene samples obviously demonstrated that all G_RGO_ and G_GCN_ on the Cu completely coalesced into a single large-area RGO and graphene hybrid film after CVD for 900 s, suggesting the scalability in this study (Supplementary Fig. S8). Similar to Fig. 2, RGO and CVD-grown graphene in the hybrid film (conformed by Raman spectroscopy, Supplementary Fig. S8) were clearly distinguished by optical microscopy due to their different thickness^37^,^38^,^39^ (Fig. 3a and Supplementary Fig. S8). The thick RGO (bluish and yellowish areas) exhibited relatively strong G and D peak intensities (I_G_ and I_D_, respectively) compared with the thin CVD-grown graphene specimens (G_RGO_ and G_GCN_) (Fig. 3b and Supplementary Figs S8 and S9). For a precise analysis, Raman maps of I_D_/I_G_ (Fig. 3c) and I_2D_/I_G_ (Fig. 3d) of the area near the thick RGO (Fig. 3a,b, yellow box) were measured. The SEM image of the same area of the RGO-graphene hybrid film sample was also presented with the coordinates denoted by x and y with subscripts (Fig. 3e). For clarity, the perimeter of the thick RGO was indicated by a green-dotted line in Fig. 3a–e. I_D_/I_G_ exhibited non-monotonous evolution when moving from the thick RGO (Fig. 3c, left end) to the high-quality monolayer graphene (Fig. 3c, right end), while I_2D_/I_G_ showed a monotonous increase (Fig. 3d). In particular, I_D_/I_G_ increased from 0.8 to 1.3 in the thick RGO region (Fig. 3c, left region near green-dotted line), whereas I_D_/I_G_ gradually decreased from 1.3 to 0.2 in the region between the green-dotted line and the white-dashed line. This region exhibited the thickness comparable that of the high-quality monolayer graphene (Fig. 3a,e). Unlike I_D_/I_G_, I_2D_/I_G_ increased monotonously from 0.2 to 2.0 when moving from the thick RGO (Fig. 3d, dark-blue region) to the high-quality monolayer graphene (Fig. 3d, dark-red region). Furthermore, as shown in Fig. 3f, when the I_2D_/I_G_ values (Fig. 3c) were shown as a function of the corresponding I_D_/I_G_ values (Fig. 3d) while moving from x_1_ to x_11_ along the x-axis at each point in the y-axis (Fig. 3e), noticeably, all the experimental data collapsed into a single curve (red-dashed line).

High-quality Raman spectra (Fig. 3g) were further recorded along the red-solid line in Fig. 3e for more details on the spatial variations of I_D_/I_G_ (Fig. 3h, black square), I_2D_/I_G_ (Fig. 3h, red circle), the 2D peak frequency (ω_2D_, Fig. 3i, green pentagon) and the 2D peak width (Γ_2D_, Fig. 3i, blue hexagon). A certain value of 2702 cm^−1^ was subtracted from all the 2D peak frequencies for convenience. According to previous studies^33^,^34^,^35^,^36^, I_D_/I_G_ of graphene evolves non-monotonously even though defect density in the graphene changes monotonously. In particular, I_D_/I_G_ increases monotonously until the defect density in graphene increases up to a certain value, whereas it decreases monotonously when the defect density higher than the certain value further increases. In contrast, the 2D peak intensity significantly decreases only when the defect density increases in the vicinity of the certain value^35^,^36^. Compared with the 2D peak intensity, the G peak intensity exhibits relatively weak variations when the defect density changes, indicating that I_2D_/I_G_ of graphene decreases monotonously with increasing defect density. Moreover, the 2D peak frequency (ω_2D_) and width (Γ_2D_) do not show a significant variation until the defect density increases up to the certain value^36^. However, ω_2D_ considerably decreases when the defect concentration further increases over the certain value, while Γ_2D_ strongly increases. As shown in Fig. 3h, I_D_/I_G_ increased until the position moved from x_1_ to x_4_, however, it decreased when the position further moved from x_4_ to x_8_ (black square). In addition, I_2D_/I_G_ (Fig. 3h, red circle) and ω_2D_ (Fig. 3i, green pentagon) increased monotonously when the position moved from x_1_ to x_8_, while Γ_2D_ (Fig. 3i, blue hexagon) decreased monotonously. These evolutionary behaviours with moving the position from x_1_ to x_8_ is good agreement with those of Raman characteristics of graphene appearing when the defect density in the graphene decreased monotonously via the certain value. The agreement suggested that the defect concentration in the graphene sample decreased with the variation of position from x_1_ to x_8_, indicating that graphene growth from RGO showed appreciable improvement in structural quality after continuous extending within a few hundred nanometres. As shown in Fig. 3c–e,h, the points in the *x*-axis at which I_D_/I_G_ and I_2D_/I_G_ no longer exhibited a discernible change tended to correspond well with the prominent dark line feature on the SEM image (Fig. 3e, white-dashed line). We proposed that the dark line feature was formed at the boundary when G_RGO_ and G_GCN_ coalesced into a single large film as a final product.

### Surface property of graphene grown from RGO

**Figure 4:**
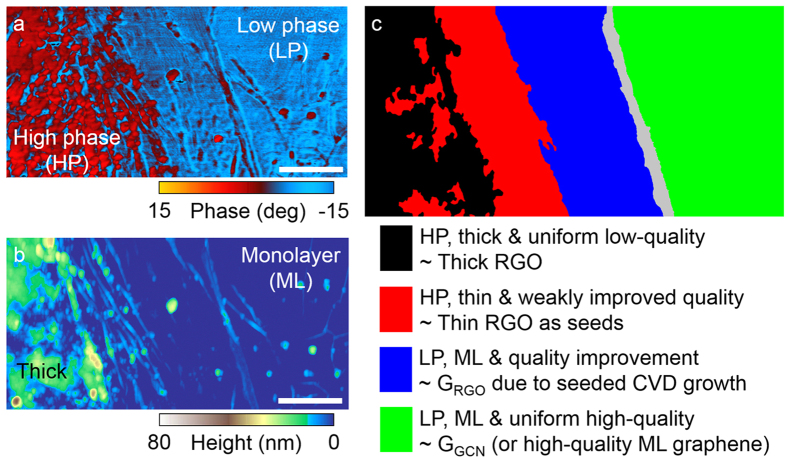
Surface characterization of graphene grown from RGO flakes. (**a**) AFM phase image of the graphene sample in Fig. 3c–e and (**b**) its corresponding AFM topographic image. The scale bars in (**a,b**) are 660 nm. (**c**) Schematic representation of regions of thick RGO, high-quality monolayer graphene, high-phase thin RGO and low-phase G_RGO_ in (**a,b**).

Although thick RGO has been clearly distinguished from thin graphene using an optical microscope (Fig. 3a), SEM (Fig. 3e) and precise spatially-revolved Raman spectroscopy (Fig. 3b–d,f–i), it is still unclear whether the thin graphene exhibiting the evolution of structural quality include a thin RGO. However, we found that RGO films were distinguished from CVD-grown monolayer graphene films using phase imaging technique^46^ based on an atomic force microscope (AFM) (Supplementary Fig. S10). In particular, the AFM phase value of the RGO surface was relatively larger than that of the CVD-grown graphene surface. To distinguish between RGO and CVD-grown graphene in the thin graphene interconnected with thick RGO, AFM phase image (Fig. 4a) and AFM topographic image (Fig. 4b) were measured over the same region in Fig. 3c–e. The AFM phase values in the thick RGO (Fig. 4c, black region) were substantially higher than those in the high-quality monolayer graphene (Fig. 4c, green region). Notably, the AFM phase values in the region (Fig. 4c, red region) adjacent to the thick RGO were largely equivalent to those of the thick RGO, although the height of this adjacent region was relatively lower than that of the thick RGO. Unlike the high-phase (HP) thin graphene, the AFM phase values in the region (Fig. 4c, blue region) between the HP thin graphene and the high-quality monolayer graphene were largely equivalent to those of the high quality monolayer graphene. The extended length of the low-phase (LP) thin graphene from HP thin graphene was approximately 800 nm. This extended length was a good agreement with the theoretically estimated growth length of G_RGO_ after CVD for 900 s (Fig. 1f). The results suggested that the HP and LP thin graphene regions were thin RGO and G_RGO_, respectively, indicating the monotonous decrement of the defect concentration with the change of position from left to right over G_RGO_ whose size was a few hundred nanometres.

## Discussion

Transmission electron microscopy (TEM) is complementary to Raman spectroscopy, ideally suited for more detailed microanalysis of graphene atomic structure and its derivatives^22^,^23^,^47^. Thus, an TEM diffraction study was further performed for graphene samples formed by CVD growth on the Cu substrate with RGO for 100 s (Supplementary Fig. S11). When the position moved from the RGO edge to the growth front of G_RGO_, the corresponding recorded selected area electron diffraction (SAED) patterns varied from fully amorphous diffraction rings to the inner diffraction spots exhibiting streaking. This TEM observation suggested that graphene showed improvement in the structural quality while it was grown from RGO on the Cu by CVD for 100 s^47^.

We have already demonstrated that G_RGO_ and G_GCN_ coalesce into a single large-area film as a RGO-graphene hybrid film (Supplementary Fig. S8). Furthermore, we investigated and compared optical and electrical properties of RGO-graphene hybrid film samples to gauge the device applicability and quality of the samples (Supplementary Fig. S12). The optical transmittance at 550 nm and the sheet resistance of the RGO-graphene hybrid film samples (see Methods) were 96.9% and 1252 Ω sq^−1^, respectively, comparable with those of high-quality monolayer graphene^2^,^48^,^49^,^50^,^51^. In addition, these values varied by controlling the density of RGO flakes on the entire RGO-graphene hybrid films. The good performance and variable characteristics suggest that our final products as large-area RGO-graphene hybrid films may be a good candidate for the device applicability such as flexible transparent electrodes in various applications.

In conclusion, we have presented an experimental study that investigates the evolution of the size and structural quality of graphene during seeded CVD growth of graphene from RGO flakes on Cu foils. In the initial stages, seeded growth of graphene from RGO on Cu surfaces was slower than simultaneous growth of typical high-quality monolayer graphene from graphene seed crystals spontaneously nucleated on the same Cu surfaces during the CVD process. Moreover, the early-grown graphene from the RGO seeds exhibited low structural quality. In the later stages, however, the growth rate of the graphene from RGO was comparable with that of the typical graphene. More noteworthy was that the graphene growth from RGO showed appreciable improvement in structural quality and completely coalesced with the typical high-quality monolayer graphene after continuous extending within a few hundred nanometres. These results suggested that seeded growth of graphene from RGO on Cu was accompanied by the efficient restoration of graphitic structure during CVD process, providing a clue for detailed understanding of the growth of large-area high-quality graphene film from low-quality seeds on the Cu during the CVD process. Therefore, the finding can serve as a route for achieving tailored large-scale graphene-based hybrid materials with required properties.

## Methods

### GO preparation

GO was arranged by the exfoliation and oxidation of natural graphite flakes (Sigma-Aldrich) according to the modification of Hummers method. A mixture of natural graphite flake (5.0 g) and NaNO_3_ (3.75 g) was added to a round-bottom flask (2,000 ml) containing H_2_SO_4_ (95%, 375 ml) while stirring in an ice bath. KMnO_4_ (22.5 g) was added slowly to keep the reaction temperature of the suspension below 20 °C. Next, the flask was placed in the oil bath at 30 °C. The oil bath was removed completely at the end of the 2 days. After air cooling, diluted H_2_SO_4_ (5%, 700 ml) was added slowly to the flask, and the stirring was maintained for 2 h. To this mixture, H_2_O_2_ (30%, 15 ml) was added slowly and then the colour of the suspension turned dark brown to yellow, and the stirring was continued for 2 h. The obtained graphite oxide was purified with distilled water repeatedly by centrifugation. GO sheets were exfoliated from graphite oxide by ultra-sonication. The products were re-dispersed in distilled water.

After exposure to UV, a surface of Cu foil turns into hydrophilic surfaces and it was dipped in mixture of toluene (100 ml) and (3-aminopropyl)triethoxysilane (3-APTES, 97%, 0.3 ml) to ensure a uniform GO distribution on the Cu foil. The 3-APTES treated Cu foil was rinsed sequentially with ethanol after toluene and dried in a 60 °C atmosphere for 20 min. At the end of the process, the Cu foil was immersed in the GO solution (1.5 mg/1 ml) for 1 min and dried at room temperature.

### Graphene synthesis and transfer

Following GO deposition onto a Cu foil (25-μm-thick, 99.8% purity, Alfa Aesar), the Cu foil was loaded into a CVD furnace and heated up to 1000 °C under a pressure of 1.0 Torr while 100 s.c.c.m. hydrogen gas (H_2_) was introduced. The growth of graphene was then performed at 1060 °C for a certain time under a gas mixture of 2 s.c.c.m. methane gas (CH_4_), diluted in 300 s.c.c.m. argon gas (Ar) and 60 s.c.c.m. of H_2_. Finally, as prepared, the sample was cooled down to room temperature with Ar and H_2_ after turning off the flow of CH_4_. After all the process was over the Cu foil was removed from the furnace for further characterization. For Raman spectroscopy and its correlated studies, the graphene samples on the Cu foil were transferred to SiO_2_/Si wafers (Si(100) covered by 300-nm-thick SiO_2_) using a poly(methyl methacrylate) (PMMA) assisted process. Briefly, the PMMA dissolved in chlorobenzene was spin-coated onto the graphene samples at 2,000 r.p.m. for 30 s. The PMMA-coated samples were placed in a Cu etchant (CE-100, Transene Company) to remove the Cu foil. After complete etching of Cu foil, the PMMA-coated samples were scooped out of the etchant using the SiO_2_/Si substrates. Finally, the PMMA layer was then removed with acetone and the surface was further rinsed several times with deionized water.

### Raman characterization

Spatially resolved Raman spectroscopy was performed using a Raman microscope (NTEGRA Spectra, NT-MDT, Moscow) at Korea Basic Science Institute, equipped with a piezoelectric sample scanner. The wavelength of the excitation laser was 473 nm, and the power of the laser was kept below 0.3 mW without noticeable sample heating. The laser spot size was approximately 0.32 μm with a 100× objective lens (numerical aperture = 0.90). The spectral resolution was 2.0 cm^−1^ (using a grating with 600 grooves mm^−1^). The intensity of each Raman peak was extracted from the maximum value without any data processing over the corresponding spectral range (1,330–1,410 cm^−1^ for the D band, 1,560–1,620 cm^−1^ for the G band and 2,670–2740 cm^−1^ for the 2D band).

## Additional Information

**How to cite this article**: Chang, S.-J. *et al.* Graphene growth from reduced graphene oxide by chemical vapour deposition: seeded growth accompanied by restoration. *Sci. Rep.* **6**, 22653; doi: 10.1038/srep22653 (2016).

## Supplementary Material

Supplementary Information
