# Supplementary material for: Graphene growth from reduced graphene oxide by chemical vapour deposition: seeded growth accompanied by restoration
Source: Sci Rep. 2016 Mar 10;6:22653. doi: 10.1038/srep22653 (PMC4785362; doi:10.1038/srep22653)
Supplement: Supplementary Information [file srep22653-s1.pdf]

Supplementary Information for

# Graphene growth from reduced graphene oxide by chemical vapour deposition: seeded growth accompanied by restoration


Sung-Jin Chang,*[,1] Moon Seop Hyun,[1,2] Sung Myung,[3] Min-A Kang,[3] Jung Ho Yoo,[2] Kyoung G. Lee,[4] Bong Gill Choi,[5] Youngji Cho,[2,6] Gaehang Lee,[7] Tae Jung Park*[,1]

[1] *Department of Chemistry, Chung-Ang University, 84 Heukseok-ro, Dongjak-gu, Seoul 06974, Republic of Korea*

[2] *Measurement & Analysis Team, National Nanofab Center, 291 Daehak-ro, Yuseong-gu, Daejeon 305-701, Republic of Korea*

[3] *Thin Film Materials Research Center, Korea Research Institute of Chemical Technology, 141 Gajeong-ro, Yuseong-gu, Daejeon 305-600, Republic of Korea*

[4] *Department of Nano Bio Research, National Nanofab Center, 291 Daehak-ro, Yuseong-gu, Daejeon 305-701, Republic of Korea*

[5] *Department of Chemical Engineering, Kangwon National University, 346 Joongang-ro, Samcheok 245-711, Republic of Korea*

[6] *Department of Applied Science, Korea Maritime and Ocean University, Busan 606-791, Republic of Korea*

[7] *Korea Basic Science Institute, 169-148 Gwahang-ro, Yuseong-gu, Daejeon 305-806, Republic of Korea*

* To whom correspondence should be addressed.

Email: sungjin.chang@gmail.com (S.-J.C.); tjpark@cau.ac.kr (T.J.P.)

## 1. Reduction of graphene oxide flakes after the CVD process

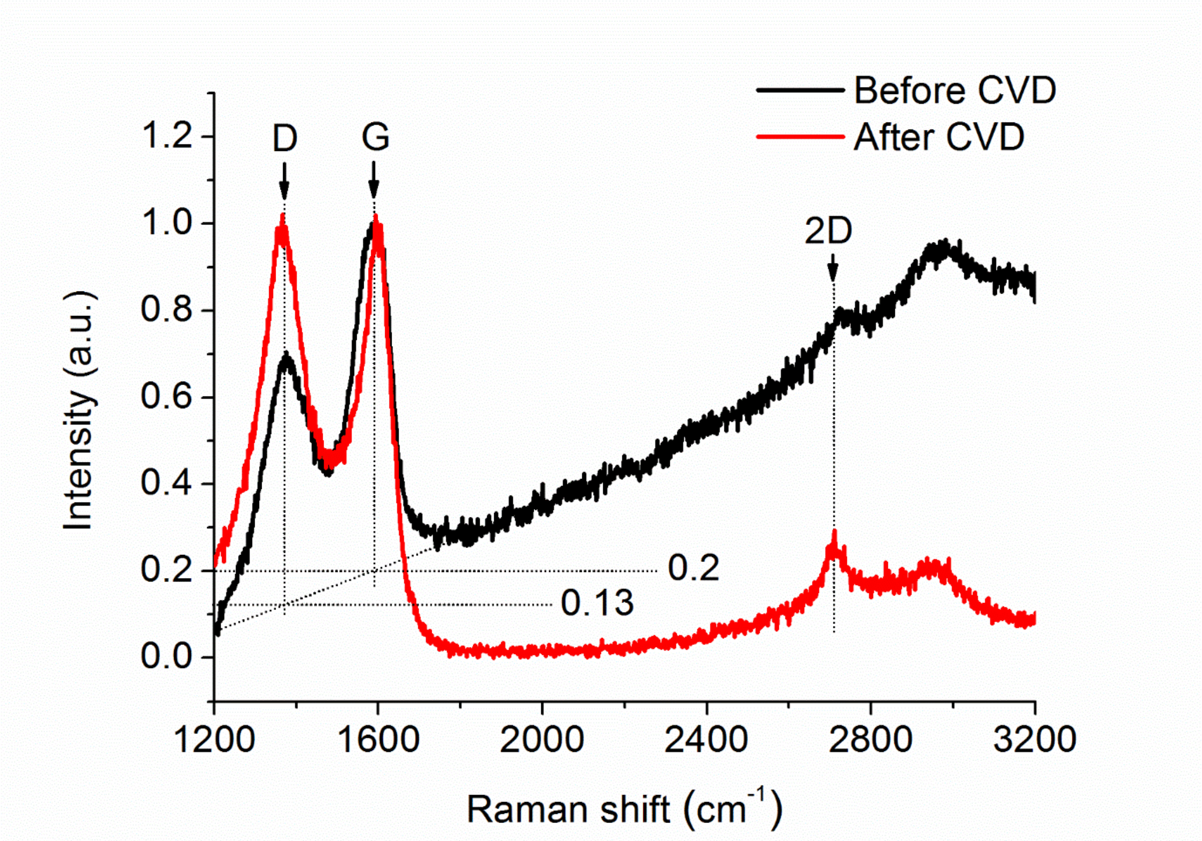


**Figure S1.** Raman spectra of graphene oxide before and after the CVD process for graphene growth.

Figure S1 presents the representative Raman spectra of graphene oxide (GO) flakes before and after CVD process for graphene growth. The ratio of the D peak intensity ($I_D$) to the G peak intensity ($I_G$) of the GO flakes after the CVD process increases even though the baseline slope of the Raman spectrum of the GO flakes before the CVD process is taken into account, indicating that the reduction process altered the structure of GO with a high quantity of structural defects[S1-S3]. The increase of the 2D peak intensity in the Raman spectrum of the GO flakes after CVD approximately at 2715 $cm^{-1}$ also indicates better graphitization compared with the GO flakes before CVD.

## 2. Raman spectroscopy of graphene grown on Cu with reduced GO (RGO) flakes by CVD

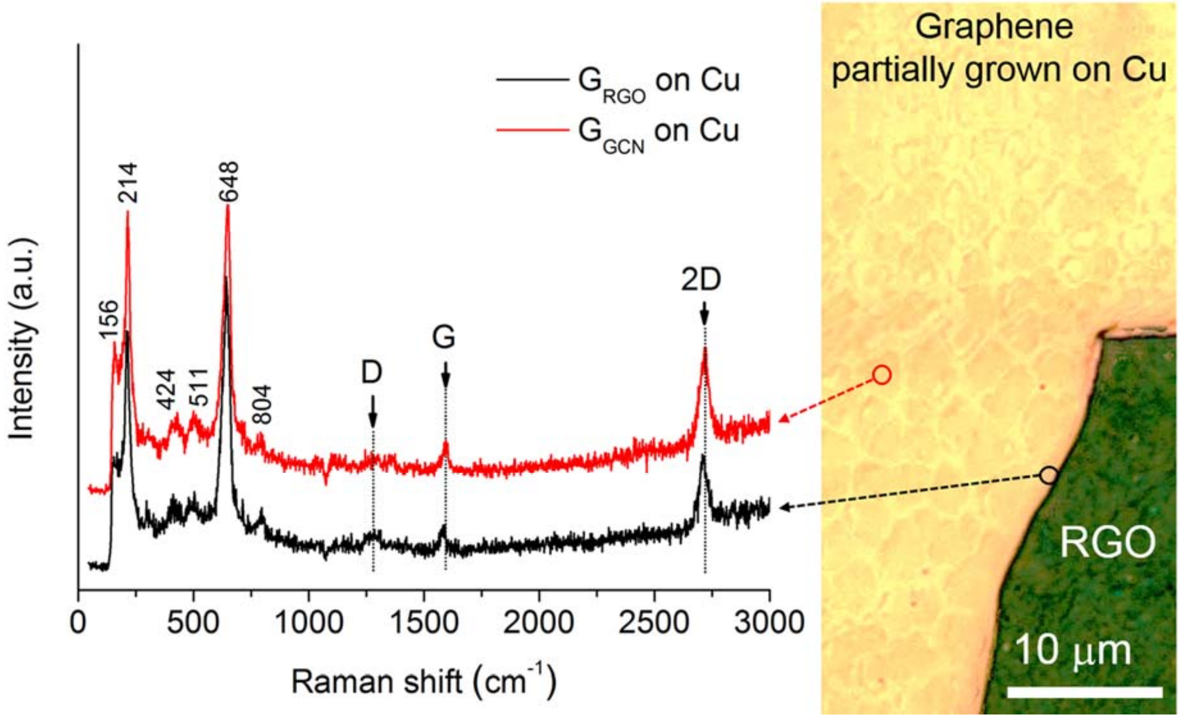


**Figure S2.** Raman spectra (left) and optical microscope image (right) of as-synthesized graphene on Cu with RGO flakes by CVD for 10 s.

The existence of the G and 2D peaks in the Raman spectra on the left side in Fig. S2 confirms that the features newly synthesized on the Cu substrate after CVD growth are graphene. Within 140–900 $cm^{-1}$ spectral region several peaks are observed. These peaks are attributed to the existence of a $Cu_2O$ underlayer[S4,S5], formed between the Cu substrate and the graphene layer.

**3. Growth of graphene islands on Cu without RGO by CVD**

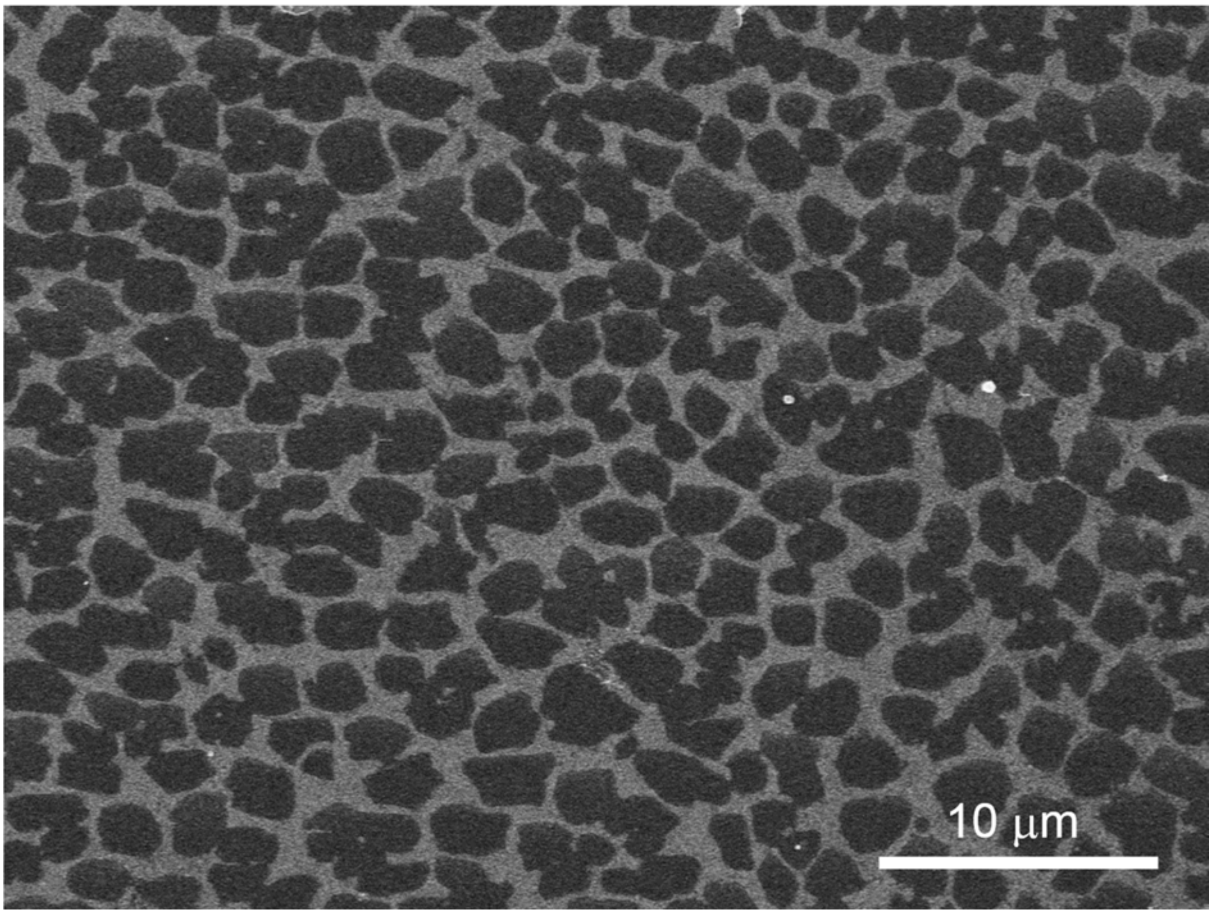


**Figure S3.** SEM image of graphene islands grown on the Cu substrate without RGO by the CVD process for 5 s.

## 4. The growth length of the graphene grown from the RGO edge

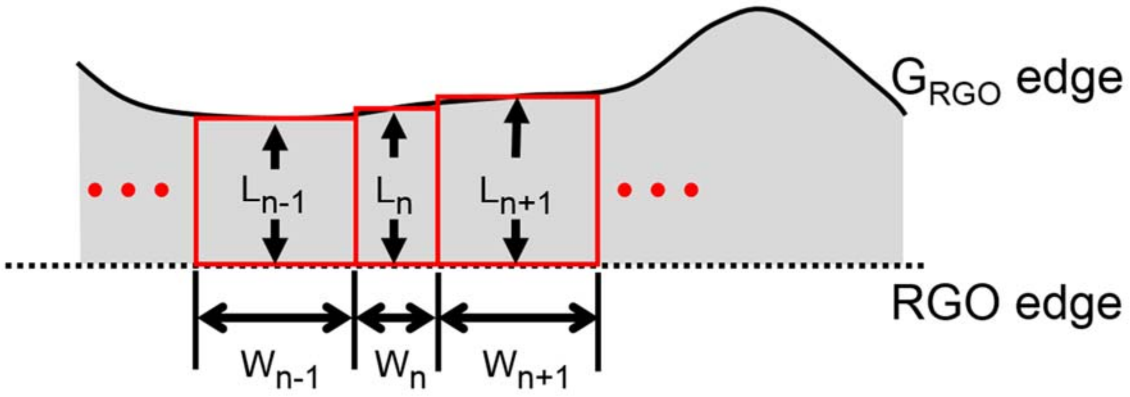


**Figure S4.** Schematic of model for obtaining the average growth length of $G_{RGO}$ from SEM image.

The average growth length of $G_{RGO}$ (=$L_{ave}$) is given by

$$\frac{\sum_n (L_n W_n)}{\sum_n W_n}$$

,
where $L_n$ and $W_n$ is defined by the length and the width of the partial $G_{RGO}$ (Fig. S4). It is worth noting that individual $W_n$ values are used as frequencies of the corresponding $L_n$ for evaluation of the average growth length of $G_{RGO}$ by converting them into integer numbers.

The standard deviation of the growth length of $G_{RGO}$ is given by

$$\sqrt{\frac{\sum_n ((L_n - L_{ave})^2 W_n)}{\sum_n W_n - 1}}$$

.

## 5. Curve fitting of the time evolution of the average growth length of $G_{RGO}$

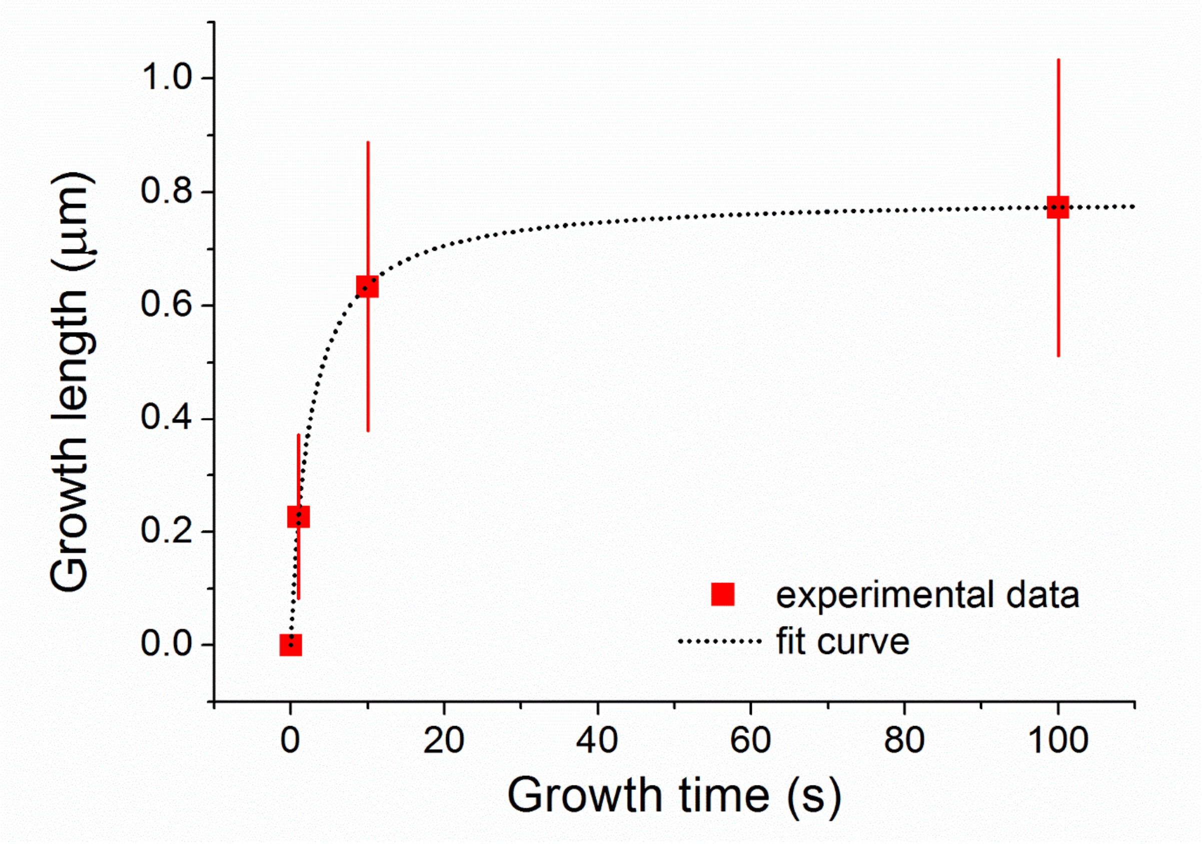


**Figure S5.** Experimental data and corresponding fit curve of the average growth length of $G_{RGO}$.

The function for obtaining fitting curve is given by

$$y = y_{max} \frac{t^n}{k^n + t^n}$$

,

where $y_{max}$, k, and n are parameters for fit curve.

**6. Growth of graphene islands on the Cu substrate with RGO by CVD**

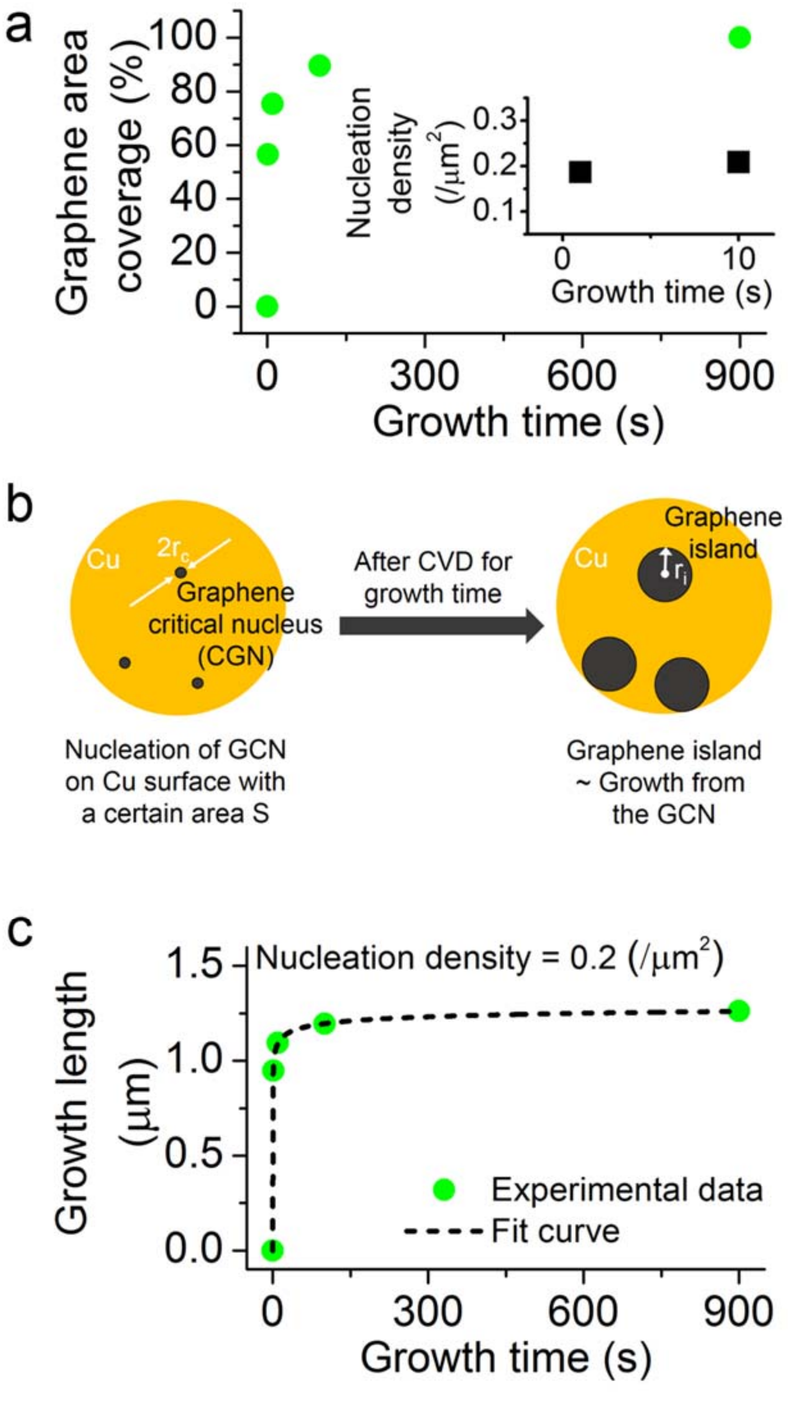


**Figure S6.** (a) Plot showing the increase of the graphene islands-covered Cu surface area with time. The nucleation density in the analyzed region of SEM image is also shown as an inset. It is worth noting that the nucleation density in SEM images of the graphene islands after CVD growth for 100 s is not available because of frequent coalescence between these individual graphene islands. (b) Schematic of a model for the growth of graphene islands on the Cu surface. (c) The average growth length of graphene grown from GCN estimated by using graphene area coverage and nucleation density on the basis of the proposed model.

Figure S6a shows graphene area coverage on the Cu substrate as a function of CVD growth time. Figure S6b schematically presents a simple model for evaluating the growth length of individual graphene islands from the results in Fig. S6a. In this model, the individual graphene islands are approximated to same circles with a radius of $r_i$. In our CVD conditions, it is impossible to directly measure the size of critical nuclei of the graphene islands because these critical nuclei very rapidly nucleate and subsequently grow during CVD growth before 1 s. Thus, it is assumed on the basis of previous studies[S6-S9] that the size of graphene critical nuclei (CGN) as carbon cluster is nearly hundred times smaller than that of the graphene islands grown after the CVD process for 1 s (approximately 930 nm radius). Under this assumption that the critical nucleus size, $2r_c$, is ignorable in comparison with $r_i$, $r_i$ can be considered as the growth length of the graphene islands (Fig. S6b). The nucleation densities are nearly comparable to each other (inset in Fig. S6a). Therefore, the graphene area coverage on the Cu is approximately given by

$$\text{Graphene area coverage}(\%) \sim \frac{\text{nucreation density} \times \pi r_i^2}{S},$$

where $r_i$ is the growth length. That is, the growth length can be estimated by using the graphene area coverage and nucleation density in Fig. S6a. Figure S6c shows the average growth length of graphene grown from GCN. Together with the experimental data, the fit curve obtained by using the function, $y=y_{max}\, t^n/(k^n+t^n)$ where $y_{max}$, k, and n are parameters for fitting curve, is also displayed in Fig. S6a as a dotted line.

## 7. Area of CVD-grown graphene near RGO on the Cu substrate

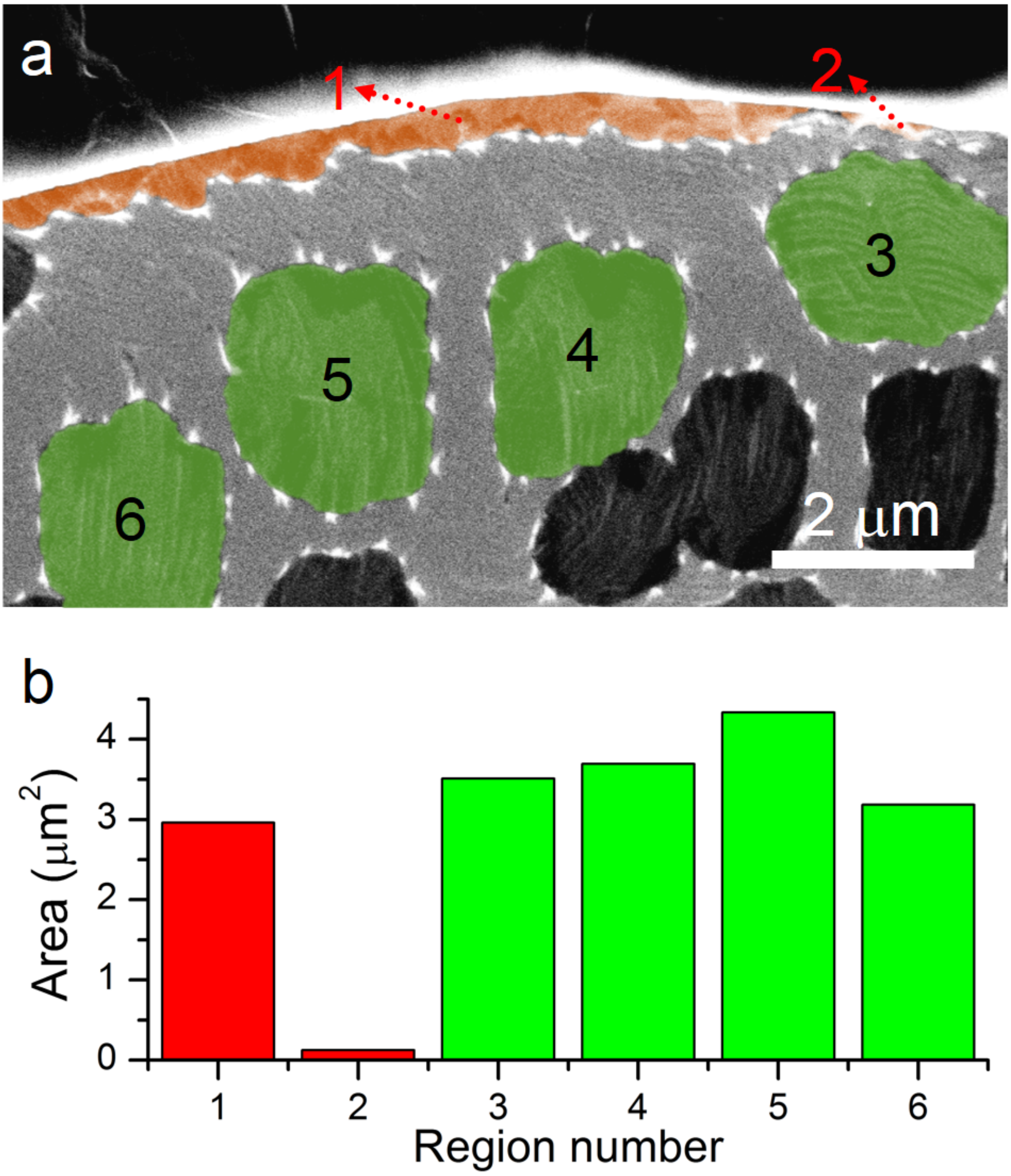


**Figure S7.** (a) SEM image of graphene regions grown near RGO on the Cu after CVD for 1 s. GRGO and their nearest GGCN are coloured as orange and green, respectively. (b) Area of the coloured graphene regions. The areas are evaluated by digital micrograph provided by Gatan program.

## 8. Large-area characterization of RGO-graphene hybrid films

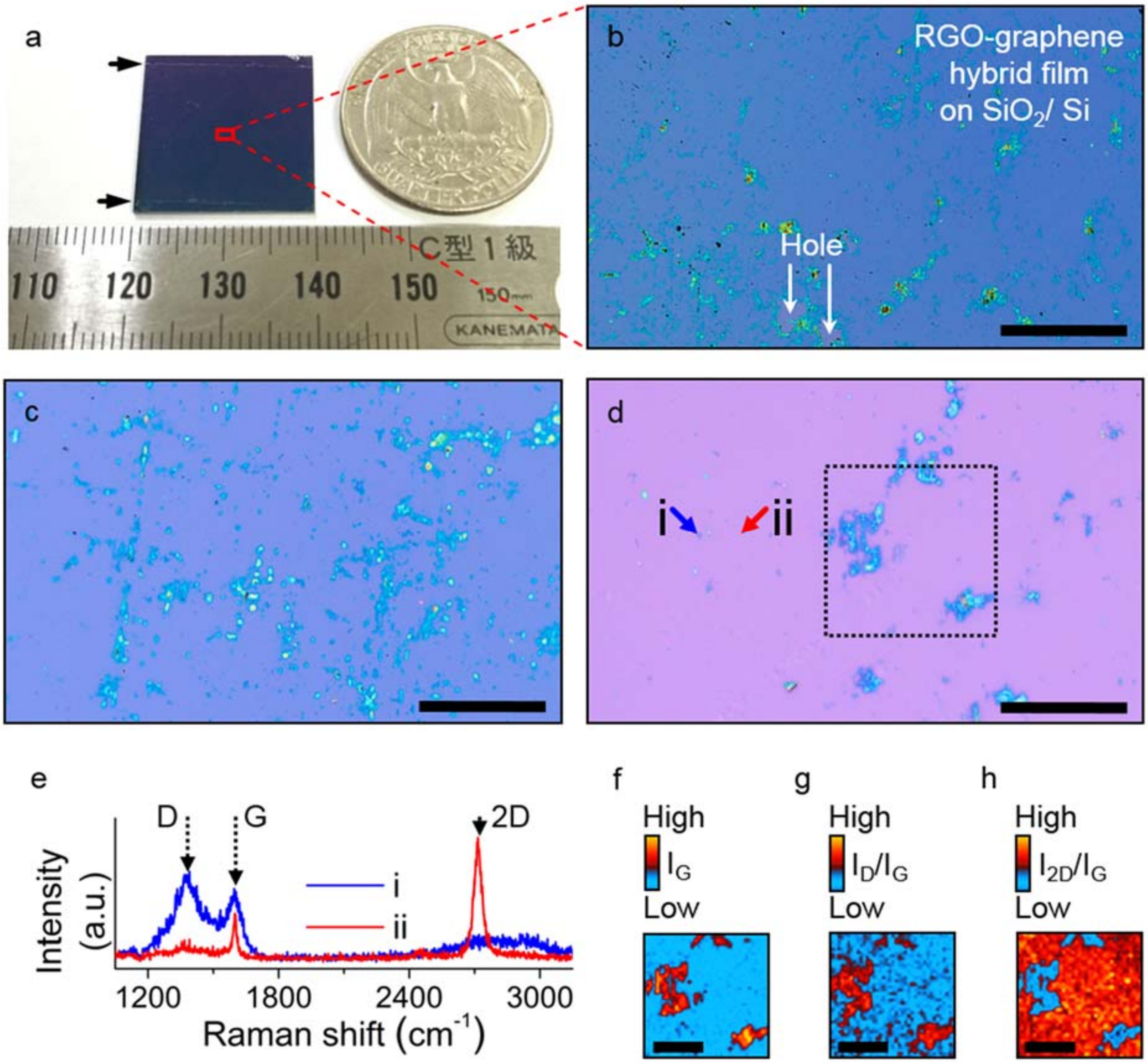


**Figure S8. Large-scale characterization of RGO-graphene hybrid films on $SiO_2$/Si.** (a) Photograph of a centimetre-sized RGO-graphene hybrid film on a $SiO_2$(300 nm)/Si substrate. (b-d) Optical microscope images of the RGO-graphene hybrid film (with increasing magnification from (b) to (d), where the scale bars are 400 μm, 100 μm, and 40 μm, respectively). (e) Representative Raman spectra of RGO flakes (blue trace) and their surrounding large-area CVD-grown graphene film (red trace). These Raman spectra of RGO and graphene were obtained from two positions, i and ii, as indicated by arrows in (d), respectively. (f-h) Raman maps of (f) the G peak intensity ($I_G$), (g) the intensity ratio of the D peak to the G peak ($I_D/I_G$) and (h) the intensity ratio of the 2D peak to the G peak ($I_{2D}/I_G$) over the same area, as indicated by dotted box in (d). The scale bars in (f-h) are 20 μm.

In order to demonstrate the scalability of this study, we characterized our samples on a large scale using optical methods (Fig. S8). Figure S8a shows a photograph of a single centimetre-sized RGO-graphene hybrid film synthesized by CVD of methane on the Cu foil with GO flakes for 900 s, and then transferred onto a $SiO_2/Si$ substrate (see Methods in main text). The hybrid film edge was discernible (Fig. S8a, white lines indicated by two arrows). The size of RGO-graphene hybrid films was only limited by the size of Cu foils and CVD chambers. Figure S8b-d shows optical microscope images of the centre of large-area RGO-graphene hybrid films on the $SiO_2/Si$ with increasing magnification from Fig. S8b to Fig. S8d. These large-area RGO-graphene hybrid films exhibit a spatially varying colour. Specifically, bluish or/and yellowish region is inhomogeneously distributed as an island in the surface of such films, and purplish region is completely covers areas between the bluish or/and yellows regions. In the surface of RGO-graphene hybrid films, holes are observed after the transfer process of the hybrid films onto the $SiO_2/Si$ (Fig. S8b). As shown in Fig. S8b, these holes are discernible because the $SiO_2/Si$ surface without the RGO-graphene hybrid film exhibits very uniform distribution of the colour whose tone is discernible in comparison with that of the $SiO_2/Si$ surface with the RGO-graphene hybrid film. In the RGO-graphene hybrid films on $SiO_2/Si$, notably, the purplish region exhibits very uniform distribution of the colour tones as compared to the bluish or/and yellowish region (Fig. S8c-d). This result indicates that the thickness of this purplish area is very uniform because spatially changing colour tones in the hybrid films are due to the spatially varying thickness of the hybrid films[S10-S12]. Raman spectroscopy confirms that the bluish or/and yellowish region and the purplish region in the surface of the RGO-graphene hybrid films are RGO and high-quality monolayer graphene[S13,S14], respectively (Fig. S8e). Furthermore, spatially-resolved Raman spectroscopy also demonstrates that high-quality monolayer graphene uniformly distributed on the (purplish) region between individual RGO flakes in the surface of the hybrid films (Fig. S8f-h), demonstrating the scalability of our study.

## 9. Raman map of the D peak intensity

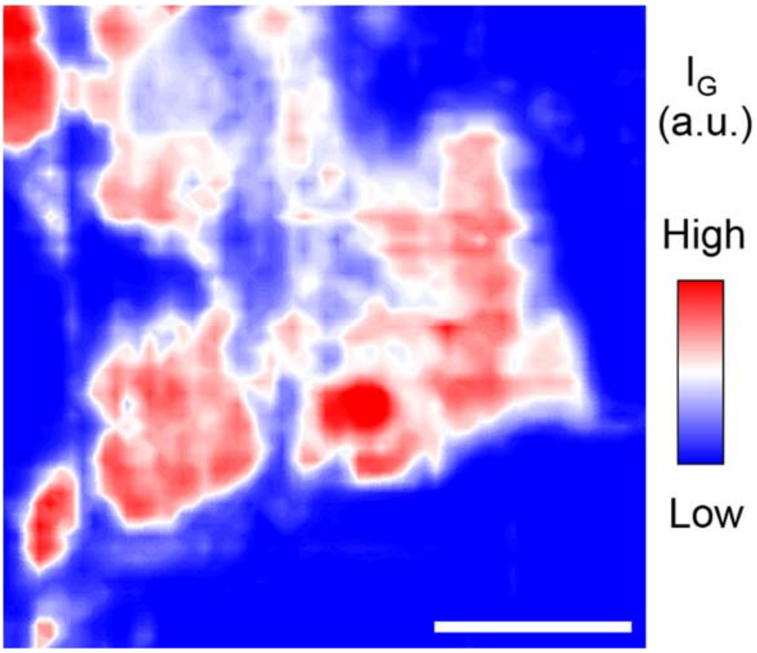


**Figure S9.** Raman map of $I_D$ of the graphene sample over the same area in Fig. 3b.

## 10. AFM phase imaging of RGO and CVD-grown graphene

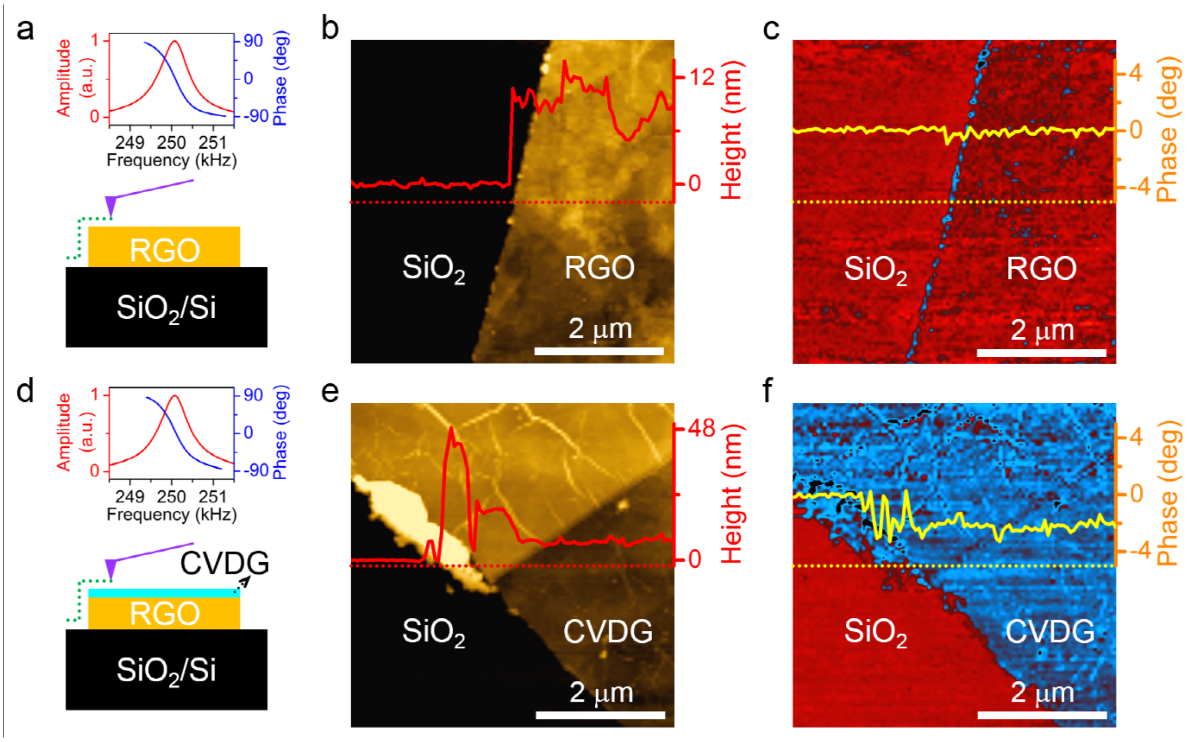


**Figure S10.** (a) Schematic of AFM measurement of RGO flakes on $SiO_2$/Si substrates. For the RGO flakes on the $SiO_2$/Si, (b) AFM topographical image and (c) its corresponding phase image. (d) Schematic of AFM measurement of CVD-grown graphene overlaying RGO flakes on the $SiO_2$/Si. For the graphene overlaying RGO flakes on the $SiO_2$/Si, (e) AFM topographical image and (f) its corresponding phase image. In (a-d), resonance characteristics of an AFM cantilever are indicated as an inset graph. In (b-c) and (e-f), the line profile along the dotted line is also presented with solid lines.

Two types of samples were prepared to demonstrate the feasibility of AFM phase imaging technique[S15] for distinguishing between surfaces of RGO and CVD-grown graphene. First, a stack of typical RGO flakes was prepared on the $SiO_2$/Si substrate (Fig. S10a), and then AFM topographical image (Fig. S10b) and its corresponding AFM phase image (Fig. S10c) of the region near the edge of the stack of RGO flakes on the $SiO_2$/Si substrate were measured. As shown in Fig. S10b,c, the AFM phase of the RGO surface is nearly the same as that of the $SiO_2$ surface, although the RGO and $SiO_2$ surfaces exhibit different morphology. Next, graphene film grown on the Cu by CVD was transferred onto the $SiO_2$/Si substrate with a stack of typical RGO flakes (Fig. S10c). It was worth noting that the edge structure in Fig. S10c was produced by scratching the top surface of the CVD-grown graphene overlaying the stack of RGO flakes on the $SiO_2$/Si substrate. Figure S10e,f shows AFM topographical image and its corresponding AFM phase image of the region near the edge of the CVD-grown graphene/the stack of RGO flakes/$SiO_2$/Si substrate structure. Unlike the RGO surface, the surface of the CVD-grown graphene exhibits the AFM phase distinct from that of the $SiO_2$ substrate. In particular, the

AFM phase value of the CVD-grown graphene surface is lower than that of the $SiO_2$ surface. Not shown here, the AFM phase value is not dependent on the existence of RGO flakes between the CVD-graphene graphene and the $SiO_2$ substrate. From these results, it can be suggested that the AFM phase value of the CVD-grown graphene surface is lower that of the RGO surface.

## 11. TEM study of graphene samples formed by seeded CVD growth on the Cu with RGO for 100 s.

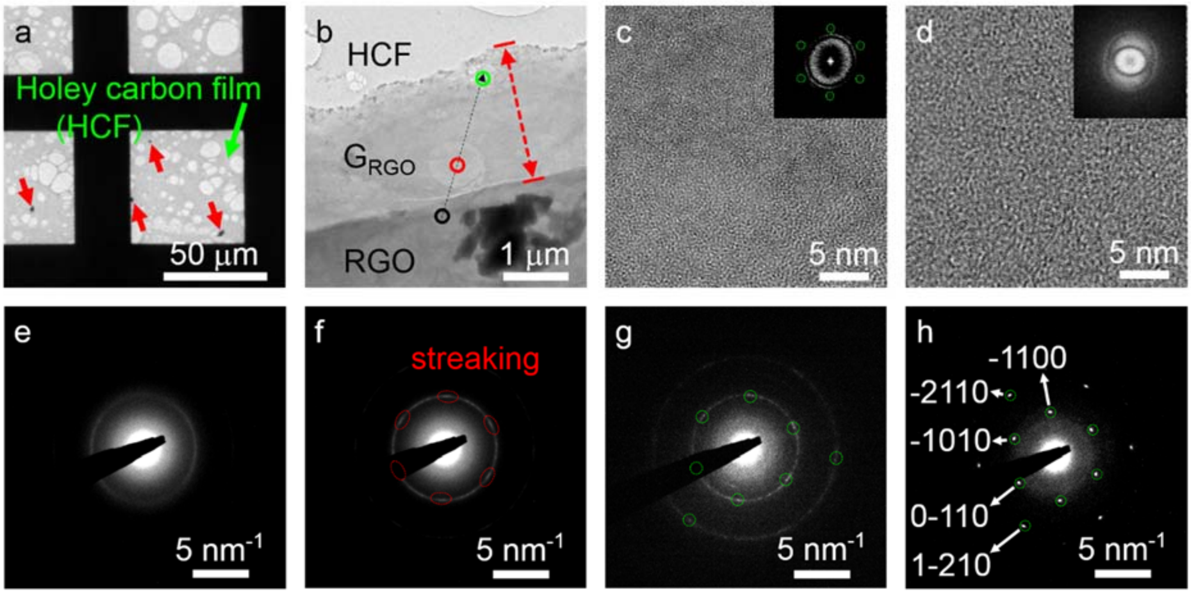


**Figure S11. Microanalysis with TEM.** (a,b) Low-magnification bright-field (BF) TEM images. (c,d) High-resolution TEM images of (c) $G_{RGO}$ and (d) RGO. (e-h) Selected area electron diffraction (SAED) patterns of (e) RGO (open black circle in (b)), (f) $G_{RGO}$ near RGO edge (open red circle in (b)), (g) $G_{RGO}$ near its growth front (open green circle in (b)) and (h) $G_{GCN}$.

We investigated the microstructure of the samples with transmission electron microscope (TEM) for more detailed microanalysis. To distinctly distinguish $G_{RGO}$ (Fig. 1e, red region) from $G_{GCN}$ (Fig. 1e, green region) in TEM observations, we used the graphene samples grown on the Cu with RGO flakes after CVD growth for 100 s (Fig. 1d) and then transferred onto TEM grid (see Methods). We examined the area near relatively thick RGO flakes to distinguish $G_{RGO}$ from RGO in TEM observations, as we did in SEM measurements (Fig. 1b-d).

RGO sheets were discernible in low-magnification bright-field (BF) TEM image of our RGO-graphene hybrid specimens (Fig. S11a, red arrows). Ribbon-shaped films thinner than the RGO sheets were also discernible near the RGO sheets in more magnified TEM image (Fig. S11b), like the case in SEM measurements (Fig. 1b-d). The width of the ribbon-shaped film (Fig. S11b, the red arrow indicates the film width) was comparable with that of $G_{RGO}$ (Fig. 1d,f), implying that the ribbon-shaped thin film was $G_{RGO}$. We could not clearly measured atomic structure of $G_{RGO}$ and RGO in high-resolution (HR) TEM images (Fig. S11c-d) as direct evidences enable to evaluate the structural properties of $G_{RGO}$ and RGO because we used the conventional FE-TEM (JEOL JEM-2100F) operated at an accelerating voltage of 200 kV for measurement of our samples. However, the inner hexagonal spots were weakly observed in the corresponded FFT pattern of the $G_{RGO}$ samples (inset in Fig. S11c) even though they were not

observed in that of the RGO samples (inset in Fig. S11d), indicating that structural quality of the $G_{RGO}$ samples was better than that of the RGO samples. Thus, we further observed selected area electron diffraction (SAED) patterns, instead of HR-TEM images, of the samples.

SAED patterns could be also used to understand the structural quality of graphene and its derivatives[S16-S18]. According to Pan *et al.*[S18], as the concentration of defects in the single-layer graphene increases up to a certain value, the outmost diffraction spots in electron diffraction pattern for the single-layer graphene exhibit streaking perpendicular to the reciprocal lattice vector, and their peak intensities decrease compared with those of the inner diffraction spots. When the concentration of defects in the single-layer graphene exceeds the certain value, the inner diffraction spots also exhibit streaking. As the defects introducing distortion into the crystal structure increase in the single-layer graphene, the intensity of all the crystalline reflection is gradually reduced and subsequently replaced by diffusing rings indicating characteristics of amorphous material. Representative SAED pattern of RGO (Fig. S11e) was similar to that of a few-layer graphene sample after a higher ion-irradiation[S18], which gave rise to fully amorphous diffraction rings due to a complete loss of long-range order within the basal planes. However, the inner hexagonal spots showing streaking were weakly observable in representative SAED patterns for $G_{RGO}$ (Fig. S11f). Moreover, blurriness of the inner hexagonal spots appreciably improved in the SAED pattern for $G_{RGO}$ near its growth front (Fig. S11g) compared with $G_{RGO}$ near the RGO edge (Fig. S11f), suggesting gradual improvement in the structural quality of $G_{RGO}$ from the RGO edge to the $G_{RGO}$ growth front. High-structural quality of $G_{GCN}$ was also confirmed by SAED pattern of $G_{GCN}$ (Fig. S11h).

## 12. Optical and electrical characterization of RGO-graphene hybrid films produced by CVD growth on Cu with RGO flakes for 900 s.

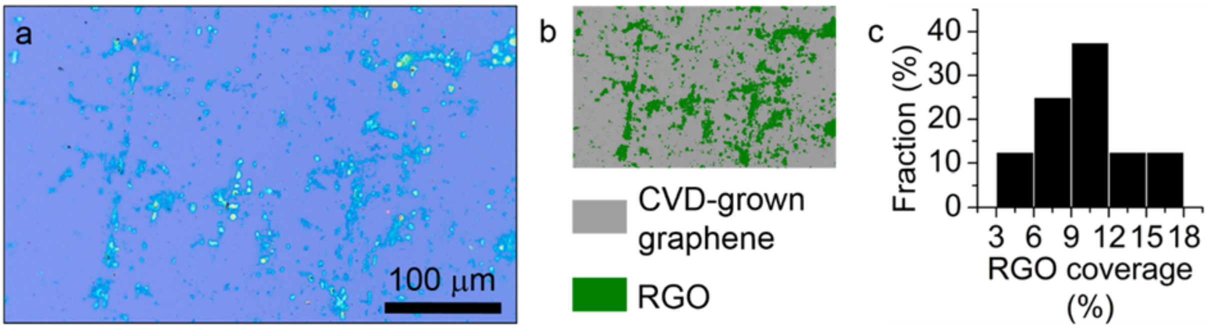


**Figure S12. RGO coverage on the surface of RGO-graphene hybrid film synthesized via CVD of methane on Cu foil immersed in the GO solution for 60 s before CVD process.** (a) Optical microscope image of RGO-graphene hybridized film on the $SiO_2$/Si. (b) Coverage of RGO and CVD-grown graphene on the hybrid film surface. (c) RGO coverage on the RGO-graphene hybrid film surface.

We further investigated the optical and electrical properties of the RGO-graphene hybrid film specimens for gauging the device applicability and quality of the samples. In particular, we prepared RGO-graphene hybrid films with low, medium and high RGO coverages using three different dipping times, 20, 60, and 600 s, respectively (see Method in the main text). Notably, RGO-graphene hybrid film samples with medium RGO coverage (Fig. S12, ~10%) were used for main experiments.

We examined visible-light transmission (Fig. S13a-b) and sheet resistance (Fig. S13b) of RGO-graphene hybrid films with three different RGO coverages to evaluate the applicability and quality of the hybrid films as transparent electrodes for touch screens and flexible displays. Note that as-synthesized hybrid films on the Cu substrate were transferred onto the polyethylene terephthalate (PET) and $SiO_2$/Si substrates for optical transmittance and sheet resistance measurements, respectively. The optical transmittances at 550 nm of the RGO-graphene hybrid films with low (~3%), medium (~10%) and high (~90%) RGO coverages were approximately 96.9 (Fig. S13a-b, black trace and circle), 94.9 (Fig. S13a-b, red trace and hexagon) and 60.8% (Fig. S13a-b, blue trace and pentagon), respectively. The sheet resistances of the RGO-graphene hybrid films with low (~3%), medium (~10%) and high (~90%) RGO coverages were approximately 1252 (Fig. S13b, black circle), 1610 (Fig. S13b, red hexagon) and 2464 $\Omega$ $sq^{-1}$ (Fig. S13b, blue pentagon), respectively. To date, indium tin oxide (ITO) films are traditionally utilized as transparent electrodes in touch panels and organic light-emitting diode panels due to the high optical transparency (~90%) and low sheet resistance (~100 $\Omega$ $sq^{-1}$)[S19].

Considering the opacity (~2.3%) and sheet resistance (~1000 Ω $sq^{-1}$) of CVD-grown monolayer graphene (Fig. R5b, green star[S20] and orange square[S21]), graphene films may be a good candidate for flexible transparent electrodes[S20]. Notably, the optical transmittances at 550 nm and sheet resistances of RGO-graphene hybrid films with low and medium RGO coverages were comparable to those of CVD-grown monolayer graphene[S20,S21]. Moreover, optical and electrical properties of RGO-graphene hybrid films were tunable with RGO coverage control to be appreciably better than those of RGO films[S22,S23]. These results indicate that our RGO-graphene hybrid films may be a good candidate for flexible transparent electrode in various applications.

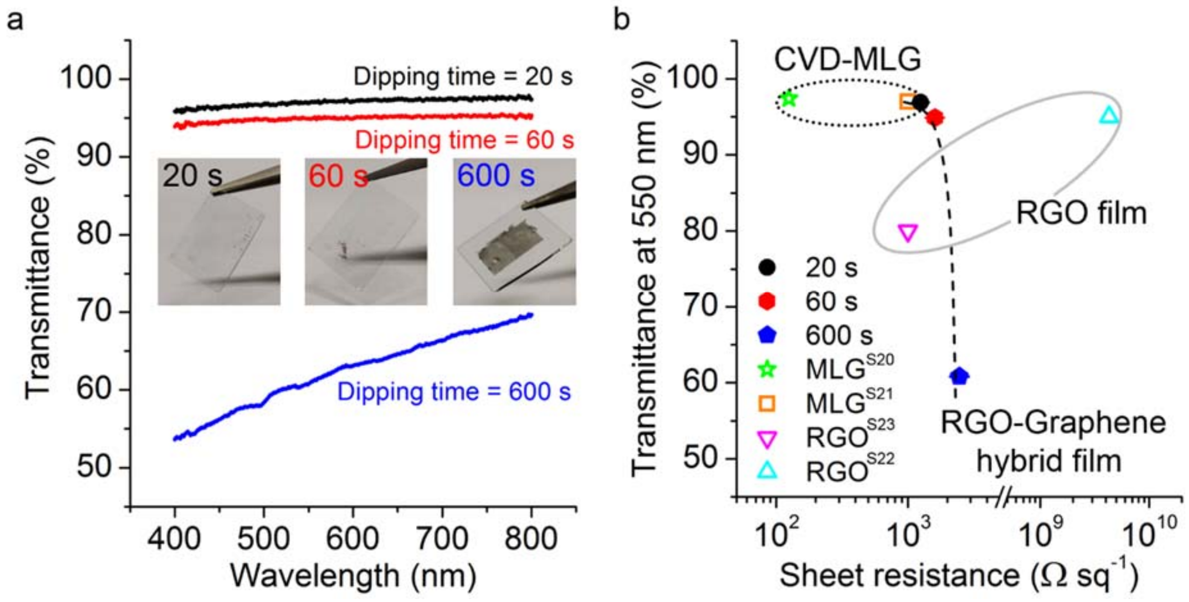


**Figure S13. Optical and electrical properties of RGO-graphene hybrid films with different RGO coverages.** (a) Optical transmittance of three types of RGO-graphene hybrid films prepared by methane CVD on Cu foil dipped in the GO solution for 20, 60, and 600 s, respectively, before the CVD process. The hybrid films prepared with different dipping times 20 s, 60 s, and 600 s exhibited different RGO coverages ~3%, ~10%, and ~90%, respectively. (b) Sheet resistance and optical transmittance at 550 nm of the RGO-graphene hybrid films. Sheet resistance and optical transmittance at 550 nm of CVD-grown monolayer graphene (MLG) and RGO films reported by several groups were also presented in (b).

## References

S1. Cançado, L. G. *et al.* General equation for the determination of the crystallite size $L_a$ of nanographite by Raman spectroscopy. *Appl. Phys. Lett.* **88**, 163106–163108 (2006).

S2. Gao, W., Alemany, L. B., Ci, L. & Ajayan, P. M. New insight into the structure and reduction of graphite oxide. *Nat. Chem.* **1**, 403–408 (2009).

S3. Yun, Y. S. *et al.* Effects of sulfur doping on graphene-based nanosheets for use as anode materials in lithium-ion batteries. *J. Power Sources* **262**, 79–85 (2015).

S4. Reimann, K. & Syassen, K. Raman scattering and photoluminescence in $Cu_2O$ under hydrostatic pressure. *Phys. Rev B.* **39**, 11113–11119 (1989).

S5. Hawaldar, R. *et al.* Large-area high-throughput synthesis of monolayer graphene sheet by hot filament thermal chemical vapor deposition. *Sci. Rep.* **2**, 682 (2012).

S6. Wang, Z. –J. *et al.* Direct observation of graphene growth and associated copper substrate dynamics by in situ scanning electron microscopy. *ACS Nano* **9**, 1506–1519 (2015). (REF. 13 in the main text)

S7. Zhang, W., Wu, P., Li, Z. & Yang, J. First–principles thermodynamics of graphene growth on Cu surfaces. *J. Phys. Chem. C* **115**, 17782–17787 (2011). (REF. 17 in the main text)

S8. Wu, B. *et al.* Equiangular hexagon-shape-controlled synthesis of graphene on copper surface. *Adv. Mater.* **23**, 3522–3525 (2011). (REF. 26 in the main text)

S9. Celebi, K. *et al.* Evolutionary kinetics of graphene formation on copper. *Nano Lett.* **13**, 967–974 (2013). (REF. 19 in the main text)

S10. R Roddaro, S. *et al.* The optical visibility of graphene: interference colors of ultrathin graphite on $SiO_2$. *Nano Lett.* **7**, 2707–2710 (2007). (REF. 37 in the main text)

S11. Ni, Z. H. *et al.* Graphene thickness determination using reflection and contrast spectroscopy. *Nano Lett.* **7**, 2758–2763 (2007). (REF. 38 in the main text)

S12. Jung, I., Rhyee, J.–S., Son, J. Y., Ruoff, R. S. & Rhee, K.–Y. Colors of graphene and graphene-oxide multilayers on various substrates. *Nanotechnology* **23**, 025708 (2012). (REF. 39 in the main text)

S13. Ferrari, A. C. *et al.* Raman spectrum of graphene and graphene layers. *Phys. Rev. Lett.* **97**, 187401 (2006). (REF. 32 in the main text)

S14. Yoon, D. *et al.* Interference effect on Raman spectrum of graphene on $SiO_2$/Si. *Phys. Rev. B* **80**, 125422 (2009). (REF. 40 in the main text)

S15. García, R., Magerle, R. & Perez, R. Nanoscale compositional mapping with gentle forces. *Nat. Mater.* **6**, 405–411 (2007). (REF. 28 in the main text)

S16. R rickson, K. *et al.* Determination of the local chemical structure of graphene oxide and reduced graphene oxide. *Adv. Mater.* **22**, 4467–4472 (2010). (REF. 22 in the main text)

S17. Gómez-Navarro, C. *et al.* Atomic structure of reduced graphene oxide. *Nano Lett.* **10**, 1144–1148 (2010). (REF. 23 in the main text)

S18. Pan, C. –T. *et al.* In-situ observation and atomic resolution imaging of the ion irradiation induced amorphisation of graphene. *Sci. Rep.* **4**, 6334 (2014). (REF. 47 in the main text)

S19. Zhu, Y., James, D. K. & Tour, J. M. New routes to graphene, graphene oxide and their related applications. *Adv. Mater.* **24**, 4924 (2012). (REF. 48 in the main text)

S20. Bae, S. *et al.* Roll-to-roll production of 30-inch graphene films for transparent electrodes. *Nat. Nanotechnol.* **5**, 574 (2010). (REF. 2 in the main text)

S21. Kim, S. H. *et al.* Carbon nanotube and graphene hybrid thin film for transparent electrodes and field effect transistors. *Adv. Mater.* **26**, 4247 (2014). (REF. 49 in the main text)

S22. Eda, G., Fanchini, G. & Chhowalla, M. Large-area ultrathin films of reduced graphene oxide as a transparent and flexible electronic material. *Nat. Mater.* **3**, 270 (2008). (REF. 50 in the main text)

S23. Becerril, H. A. *et al.* Evaluation of solution-processed reduced graphene oxide films as transparent conductors. *ACS Nano.* **2**, 463 (2008). (REF. 51 in the main text)
